# Supplementary material for: The interactome of intact mitochondria by cross-linking mass spectrometry provides evidence for coexisting respiratory supercomplexes
Source: Mol Cell Proteomics. 2017 Dec 8;17(2):216–32. doi: 10.1074/mcp.RA117.000470 (PMC5795388; doi:10.1074/mcp.RA117.000470)
Supplement: Supplemental Data [file supp_RA117.000470_133922_0_supp_23978_fzffwf.zip › spectra_annotation/mito_spectra_annotation/376-1-12-1-15-1.pdf]

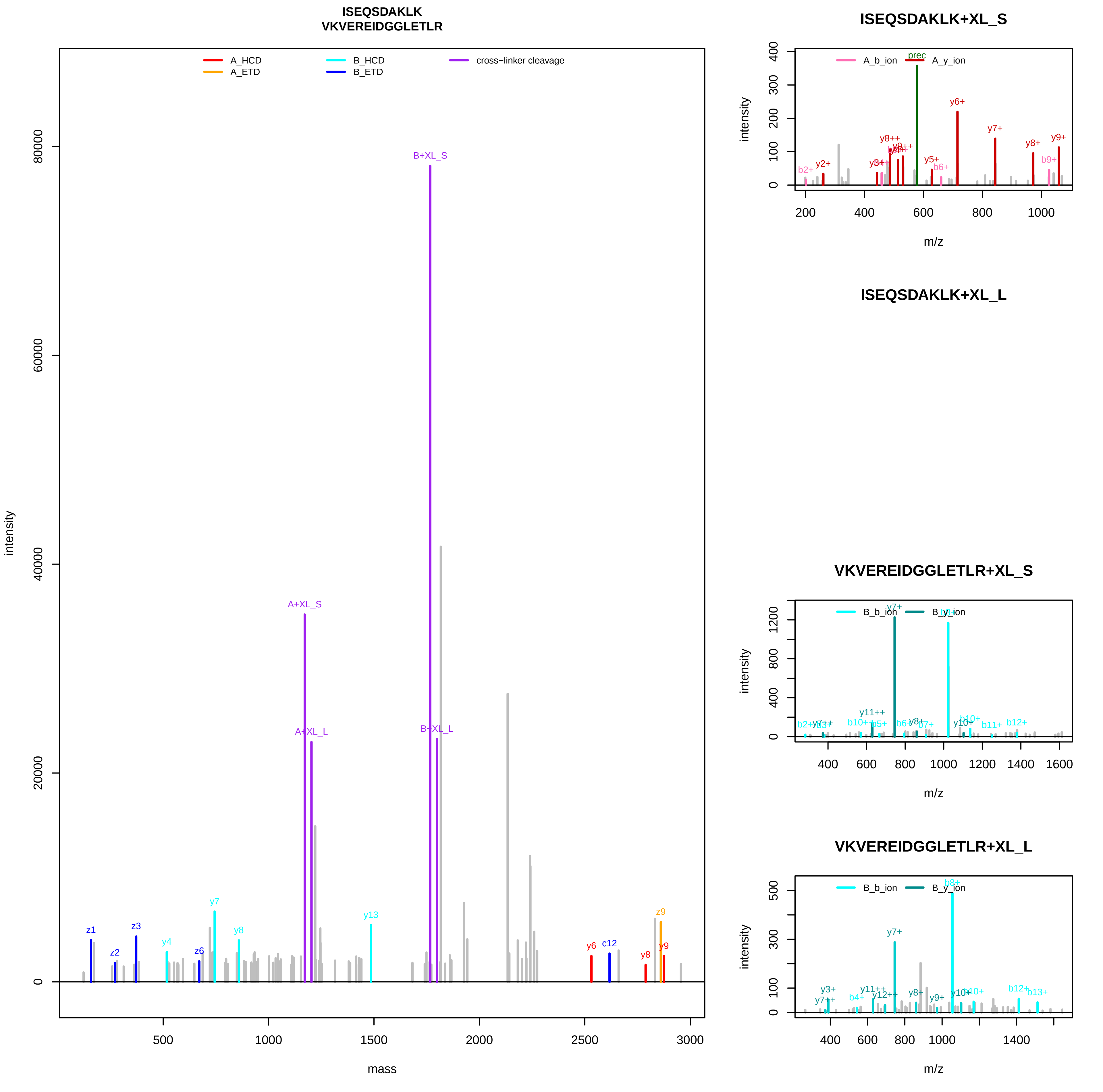
ISEQSDAKLK
VKVEREIDGGLETLR
A_HCD
A_ETD
B_HCD
B_ETD
cross−linker cleavage
B+XL_S
A+XL_S
A+XL_L
B+XL_L
z1
z2
z3
y4
z6
y7
y8
y13
y6
c12
y8
z9
y9
intensity
mass
0
20000
40000
60000
80000
500
1000
1500
2000
2500
3000
ISEQSDAKLK+XL_S
A_b_ion
A_y_ion
prec
b2+
y2+
y3+
y8++
y9++
y4+
y5+
b6+
y6+
y7+
y8+
b9+
y9+
intensity
m/z
ISEQSDAKLK+XL_L
VKVEREIDGGLETLR+XL_S
B_b_ion
B_y_ion
y7+
b9+
y11++
b10++
b5+
b6+
y8+
b7+
y10+
b10+
b11+
b12+
b2+
y7++
intensity
m/z
VKVEREIDGGLETLR+XL_L
B_b_ion
B_y_ion
b8+
y7+
y3+
y7++
b4+
y11++
y12++
y8+
y9+
y10+
b10+
b12+
b13+
intensity
m/z
